# The “WWHow” Concept for Prospective Categorization of Post-operative Severity Assessment in Mice and Rats

**DOI:** 10.3389/fvets.2022.841431

**Published:** 2022-03-15

**Authors:** Anke Tappe-Theodor, Claudia Pitzer, Lars Lewejohann, Paulin Jirkof, Katja Siegeler, Astra Segelcke, Natascha Drude, Bruno Pradier, Esther Pogatzki-Zahn, Britta Hollinderbäumer, Daniel Segelcke

**Affiliations:** ^1^Institute of Pharmacology, University of Heidelberg, Heidelberg, Germany; ^2^Interdisciplinary Neurobehavioral Core, University of Heidelberg, Heidelberg, Germany; ^3^Institute of Animal Welfare, Animal Behavior and Laboratory Animal Science, Freie Universität Berlin, Berlin, Germany; ^4^German Federal Institute for Risk Assessment (BfR), German Center for the Protection of Laboratory Animals (Bf3R), Berlin, Germany; ^5^Office for Animal Welfare and 3Rs, University of Zurich, Zurich, Switzerland; ^6^Department of Work and Environmental Protection, Westphalian Wilhelms University Muenster, Münster, Germany; ^7^Independent Researcher, Herne, Germany; ^8^Berlin Institute of Health (BIH) at Charité, QUEST Center for Responsible Research, Berlin, Germany; ^9^Department of Anesthesiology, Intensive Care and Pain Medicine, University Hospital Muenster, Münster, Germany; ^10^Member of Working Group for Animal Welfare, BÜNDNIS 90/DIE GRÜNEN, Düsseldorf, Germany

**Keywords:** postoperative, surgery surgical procedures, severity assessment, rodents, prospective, mice, rats

## Abstract

The prospective severity assessment in animal experiments in the categories' non-recovery, mild, moderate, and severe is part of each approval process and serves to estimate the harm/benefit. Harms are essential for evaluating ethical justifiability, and on the other hand, they may represent confounders and effect modifiers within an experiment. Catalogs and guidelines provide a way to assess the experimental severity prospectively but are limited in adaptation due to their nature of representing particular examples without clear explanations of the assessment strategies. To provide more flexibility for current and future practices, we developed the modular Where-What-How (WWHow) concept, which applies findings from pre-clinical studies using surgical-induced pain models in mice and rats to provide a prospective severity assessment. The WWHow concept integrates intra-operative characteristics for predicting the maximum expected severity of surgical procedures. The assessed severity categorization is mainly congruent with examples in established catalogs; however, because the WWHow concept is based on anatomical location, detailed analysis of the tissue trauma and other intra-operative characteristics, it enables refinement actions, provides the basis for a fact-based dialogue with authority officials and other stakeholders, and helps to identify confounder factors of study findings.

## Introduction

Pre-clinical animal research constitutes an essential part of several avenues to understand mechanisms and develop novel treatment options and strategies in diverse research fields. Although intense efforts are being made to replace and reduce animal experiments, they are not yet entirely dispensable. Depending on the research question, animal experimentation can be associated with harm, including pain, suffering, and distress for the animal. According to the Directive 2010/63/EU on the protection of animals used for scientific purposes, part of each application is the prospective evaluation of the severity assessment of each animal experiment in the categories of non-recovery, mild, moderate, or severe, allowing ethical consideration with respect to points weighting the likely harms to the animals against potential benefits of the planned experiments (harm/benefit analysis) (1). Therefore, researchers are required to classify the severity of every single intervention (e.g., surgical procedure, behavior test) and provide an ethical classification of the entire experiment. However, suitable categorization tools are still lacking. Besides a few reports addressing the severity classification (2, 3), three catalogs are available and widely accepted in Europe. The Berlin Animal Welfare officer catalog, named “Berlin catalog”, the EU Directive 2010/63/EU Annex VIII, called the “EU catalog” and the Swiss Federal office catalog named the “Swiss catalog” from here on (4–6). While catalogs are valuable for established interventions for which severity has been carefully evaluated in the past, they are of limited use for experiments with unknown/novel, unevaluated procedures. They can provide only rough indications of severity for comparable interventions. Furthermore, it is often not apparent which variables were used to classify individual interventions, and finally, the “nature of pain” is not sufficiently described. Nevertheless, the explicit description of the “nature of pain” is a requirement of the EU directive. Thus, a basic set of tools is needed for a prospective, transparent and objective severity assessment.

These much-needed tools should integrate the anatomical, physiological, and ethological (including evolutionary) traits of the animal species used and incorporate the specific intervention, enabling a precise, individualized prospective categorization.

The severity classification for weighing up an animal experiment is essential in many aspects. First, it allows the definition of humane endpoints and interventions to minimize the animal burden within the experiment. Second, it is of utmost importance for the experimenter to accurately predict the severity to identify direct and indirect consequences that may affect the scientific work. For example, inadequately treated pain has divergent effects, such as alterations in metabolism, hormonal imbalances (7) and psychological distress, which might also cause physiological coping mechanisms and, therefore potential confounders. Knowledge of the potential harms affects experimental design and study results. Finally, it helps to implement actions that directly or indirectly reduce the severity, maintain animal health and welfare, and minimize factors (e.g., confounder, effect modifier) related to the well-being of the individual animal, consequently increasing the quality of research.

Pain, suffering, and distress to the animal are difficult to objectify and have many different causes. The characterization of morphological tissue damage in combination with ethological parameters and methodological aspects can serve to objectify pain and suffering in surgical procedures to some extent, as they are quantifiable and interpretable parameters. For objective quantifiability, the required parameters must not be obtained purely by analogy to humans but from pre-clinical, experimental animal pain models. Over the past 25 years, several surgical pain models—primarily in rodents (rats and mice)—have been published to reveal underlying mechanisms of post-operative pain in humans (8, 9). The findings of these studies can be applied to improve clinical treatment options but also bidirectionally in animal welfare science. In our opinion, accurate knowledge of the possible pain modalities, their time courses, intensity, and localization, as well as the underlying mechanisms based on studies with pain models, can be used to provide a prospective severity assessment of surgical procedures in rodents used for scientific purposes.

Surgical interventions are ubiquitously used in rodent biomedical research for generating disease states, sampling tissue, implanting, or testing medical devices. However, it is worth pointing out that there is a specific manifestation of post-operative pain-related behavior in intensity and time dependence on the surgical characteristics (10–12).

We aimed to provide an easy-to-use concept based on intra-operative features, being orientated and adapted from different rodent models for surgically induced pain. The integration of intra-operative characteristics related to the surgical site (Where), tissue trauma (What), and methodological aspects (How) result in a method that is ready to understand, transparent in the approach, transferable to any laboratory, and applicable for any rodent surgical intervention. Thereby, the Where, What, and How (WWHow) concept enables an objective and customized prospective severity assessment of surgical interventions according to the EU directive (1).

## Methodologies For the WWHow Concept

A multidisciplinary and interprofessional group developed this concept with diverse interests and expertise in animal welfare research. These include medical professionals (veterinary and human), biologists, pre-clinical scientists, animal welfare-associated experts, and politicians (see individual affiliations). The WWHow concept is based on ordinal scales to integrate multidimensional variables into a score with an unknown interval property and relative rank of variables. Score summation for the categories “Where”, “What” and “How” gives the total score, which forms the trichotomous outcome with an ordering to the categories into mild, moderate, or severe, as suggested by the current EU directive (1). The categorization is according to the intra-operative characteristics of surgical interventions; therefore, an obvious prerequisite is to know the exact procedure.

We have chosen a score from 1 to 5 for the “Where” part and 1–9 for the “What” and “How” parts. These numbers are not mathematically consecutive; the scoring increases with the importance of the region, the size of the intervention and the duration of the surgical procedure, but not in calculative numbers, which means that a value of four does not mean double of two. The parameters considered in the establishment of the score are explained below. According to our definition, total scores between 4 and 9 points lead to a mild severity, 10–16 points belong to the moderate, and 17–23 points indicate a severe category. However, it should be borne in mind that the proposed concept is not a set of rules fixed for all time but should always be seen as a process of further development. In this context, the transition from numerical to the ordinal scale indicated here may well be modified based on future scientific results. Significantly, this categorization concept is based on one and not multiple surgical injury regions. Noteworthy, our concept is designed for mice and rats and should be adapted for other rodents.

### Intra-Operative Characteristics

#### Anatomical Localization of the Surgical Procedure (“Where”)

**Figure 1:**
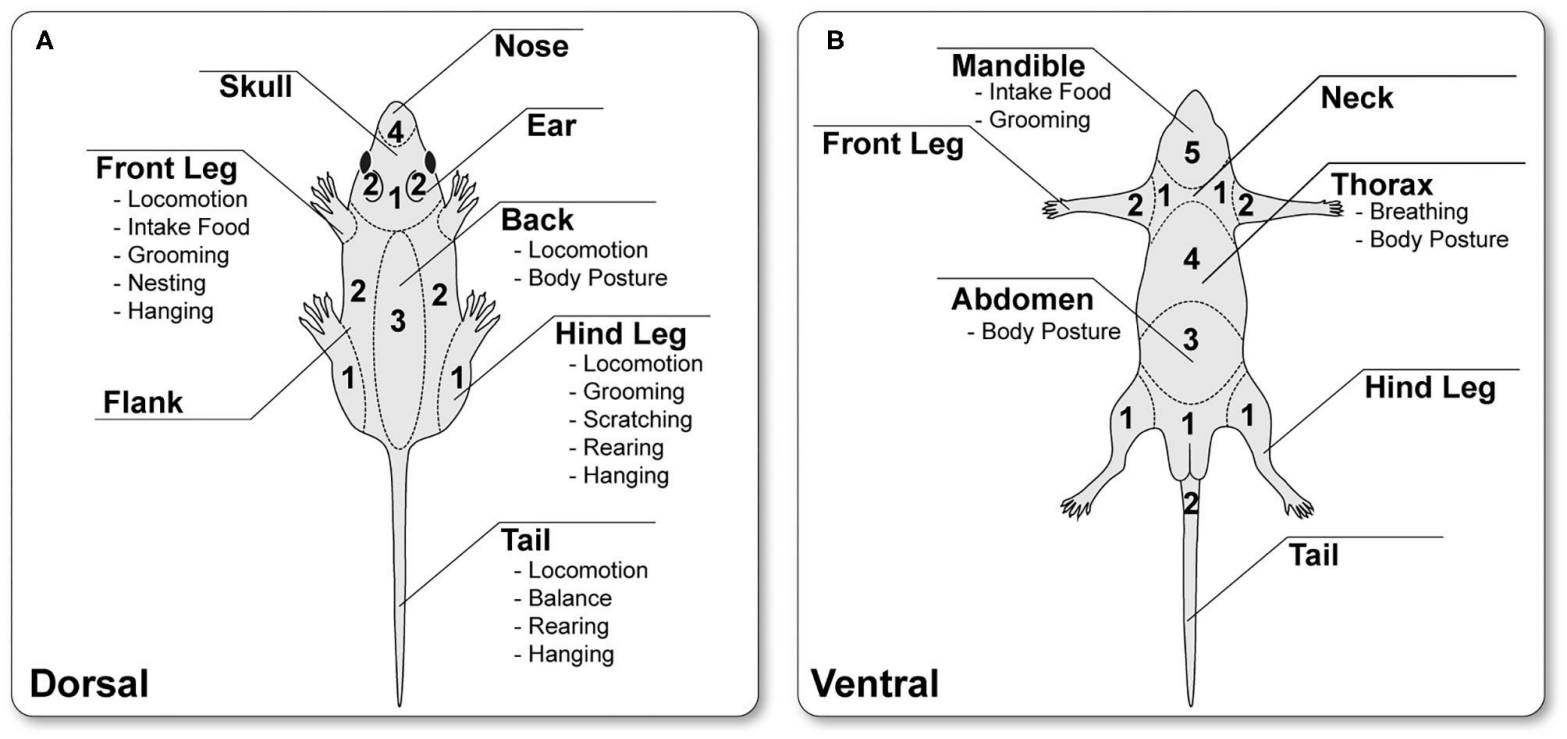
Schematic classification of the dorsal **(A)** and ventral **(B)** body surface into 11 different regions, indicated by name and particular biomechanical functions, e.g., *movement* or *maintenance* behaviors. The individual body regions are scored between 1 and 5 based on their relevance for the rodent physiological and maintenance. (The illustration was created by modifying images purchased in the PPT Drawing Toolkits-BIOLOGY Bundle from Motifolio, Inc.).

The rodent body surface was divided into 11 general regions, spanning the dorsal and ventral bodyside, including three shared regions (tail, front and hind legs) (Figure 1). The classification was based on rats' and mice's general anatomy and myology (13–16). Scoring of each body region was based on two variables, (1) biomechanical functioning and (2) its involvement in rodent-specific *maintenance and general* behavior. *Maintenance* behaviors include necessary behaviors for preserving the body and social homeostases, such as *drinking, feeding, grooming, social interaction*, and *nest building*. In contrast, *general* behaviors refer to other movement-related activities, such as exploratory or miscellaneous activities (e.g., climbing, rearing). Therefore, *maintenance* behaviors are directly linked to animal survival and are more important than *general* activity when evaluating body regions.

All extremities are involved in *movement* behaviors, including *locomotion, balancing, rearing, grooming, and scratching*. However, movement is usually not substantially restricted because rodents are quadrupeds and can compensate for the impairment, especially for an injury to the hind legs (16, 17). In contrast, unilateral injury to a front extremity leads to a more significant restriction. Here, the *food intake and* thus indirectly the *maintenance behavio*r is affected. Therefore, on an ordinal scale, the hind legs are rated 1 and the front legs 2 because of their different effects on *maintenance behavior*.

The tail, neck, flanks, back, thorax, and abdomen are essential for general *body stability in rest and* under *movement*, including *locomotion, grooming*, and *scratching*. However, the proportion of these body regions in their function is to be weighed separately; thus, the tail, the neck, and the flanks are rated with a score of 2 because they are essential for the body's stability and posture, but they are not (or less) necessary for *maintenance* behavior. The back and the abdomen are rated 3. This is due to the fact that the back and the associated muscles are involved in the body's stability and *movement* behavior. The abdomen is also necessary for this behavior and forms the body cavity for various inner organs (13, 17). The thorax, scored with 4, is particularly important because it is essential for *maintenance* (breathing) and *general* behaviors. Respiration leads to a continuous thorax movement without compensation and/or avoidance opportunities for the rodent. Rodent ears and nose (including vibrissae/whiskers) detect sounds and olfactory cues to perceive environmental changes and provide essential social communication and orientation. Therefore, the ears are rated 2, and the nose/whisker region 3. For *maintenance* behavior and ensuring survival, water and food intake are necessary. For this, the ventral part of the skull is essential for water and food ingestion and is, therefore, to be rated with a score of 5. In contrast, the dorsal cranial and genital regions are rated with 1 because surgical interventions in these regions have less impact on rodent behaviors.

#### Tissue Trauma (“What”) Caused by Surgical Procedure (“How”)

Based on findings from surgical-induced pain models in rodents, the “What” of tissue trauma and the “How” in the form of surgical techniques, i.e., size of the injury, total surgery duration, and retraction duration, may influence the type, intensity, and time course of post-operative pain and, thus, the overall severity. Therefore, these different variables of the post-operative period were integrated to form another ordinal scale for the two remaining categories. In addition, mechanistic, anatomical, and morphological data were considered. The following describes the respective variables and their potential impact in detail.

Every surgical intervention causes typical tissue trauma, which must be described as detailed as possible when assessing post-operative severity. The correlation between tissue trauma characteristics and post-operative pain-related symptoms in well-characterized procedures can help to allow a more accurate prospective assessment of surgical severity in terms of pain modalities in future procedures. One of the most critical factors for the prospective estimation of surgical-induced severity in rodents is pain, which is measurable as non-evoked or evoked pain-related behavior to different modalities (18). In addition, pain modalities are measured in pre-clinical surgical rodent pain models over time (11, 19). These evidence-based trajectories provide another essential basis for a prospective severity assessment while also identifying mechanisms that underlie different pain modalities. For example, physiological changes, e.g., in sleep behavior or stress hormone levels, are caused by pain. However, these changes are assessed sporadically in pre-clinical animal pain research so that the concept is based exclusively on the observed post-operative pain behavior, which determines the score. Overall, detailed knowledge about different pain modalities is of enormous importance for further post-operative treatment recommendations.

Most surgical procedures start with a localized skin incision. Rodent skin is thin (25 μm) with 2 or 3 layers and loose (20, 21). Cutaneous incision injury directly activates peripheral nociceptive fibers (high-threshold) and causes hemorrhage and cell debris (22). Incisions of skin tissue during surgery represent a primary wound (23). In contrast to other types of wounds (secondary or tertiary), they are characterized by fresh, aseptic injuries that have smooth edges and are closed by suturing (23). The inflammatory processes are diverse and trigger a prolonged hypersensitivity to evoked mechanical and heat but not to cold stimuli (24), directly around the incision injury (primary area of hypersensitivity). The primary hypersensitivity reaches a maximum shortly after awakening from anesthesia and is steadily reduced in the course of primary wound healing (depending on incision dimension) (10, 11).

In contrast to primary hypersensitivity, driven by peripheral processes mainly in the injured tissue, secondary hypersensitivity is a central product only to mechanical stimuli in a larger area around the incisional wound (22, 25). Peripheral sensitized nociceptors contribute to the sensitization of spinal dorsal horn neurons, expanding their receptive fields and modulating their responsiveness (22, 26). These symptoms after skin incision may manifest in avoidance/guarding behavior (27). Generally, a pure skin incision is rated 1. The type and severity of behavioral changes depend on the surgical injury location (see “Where”) as well as on the size of the injury (see “How”). Avoidance behavior is characterized by reduced weight-bearing (protective behavior) of the affected area and possibly an altered gait pattern if body regions are injured that are important for *movement* (see “Where”) (19, 28, 29). If the traumatized region is not essential for *rearing, grooming, scratching*, or *locomotion*, observations to estimate severity are hampered and can usually only be assessed by specific behavioral tests or by interpolation of mechanistic data from rodents.

Furthermore, the incised skin, most surgical procedures involve manipulating the underlying muscles, such as creating subcutaneous cavities for implantation of mini-pumps, displaying blood vessels or nerves, or providing access to internal organs (see examples in the results part). Manipulation of the muscle layer ranges from blunt dissection or displacement (30) to muscle incision (31). Unlike displacement and blunt preparations, incision always results in hemorrhage, direct activation of nociceptive fibers, and a distinct inflammatory response triggered by hemorrhage and cellular debris. These processes lead to the release of diverse damage-associated mediators, reactive oxygen species, pro-inflammatory mediators, activate residents, and facilitate the migration of immune cells, thereby altering the local tissue pH and further signaling cascades, including nociceptor sensitization (10, 32). Compared to skin-only incision, these effects are massive and, thus, have a more pronounced influence on pain behavior (31), especially during muscle contraction for *movement* (33). In contrast, blunt dissection and displacement of the muscle layers are associated with a lower degree of hemorrhage or cells debris. In addition, temporary hypoxia and mechanical stretching of the muscle play an essential role, especially in a time-dependent manner. Here, too, an inflammatory process is initiated postoperatively and is less severe and shorter than in the case of muscle injury by incision (30). Due to different degrees of inflammatory responses caused by manipulation characteristics of muscle, displacing the muscle and/or blunt dissection is rated as 1 and the muscle incision as 2.

**Figure 2:**
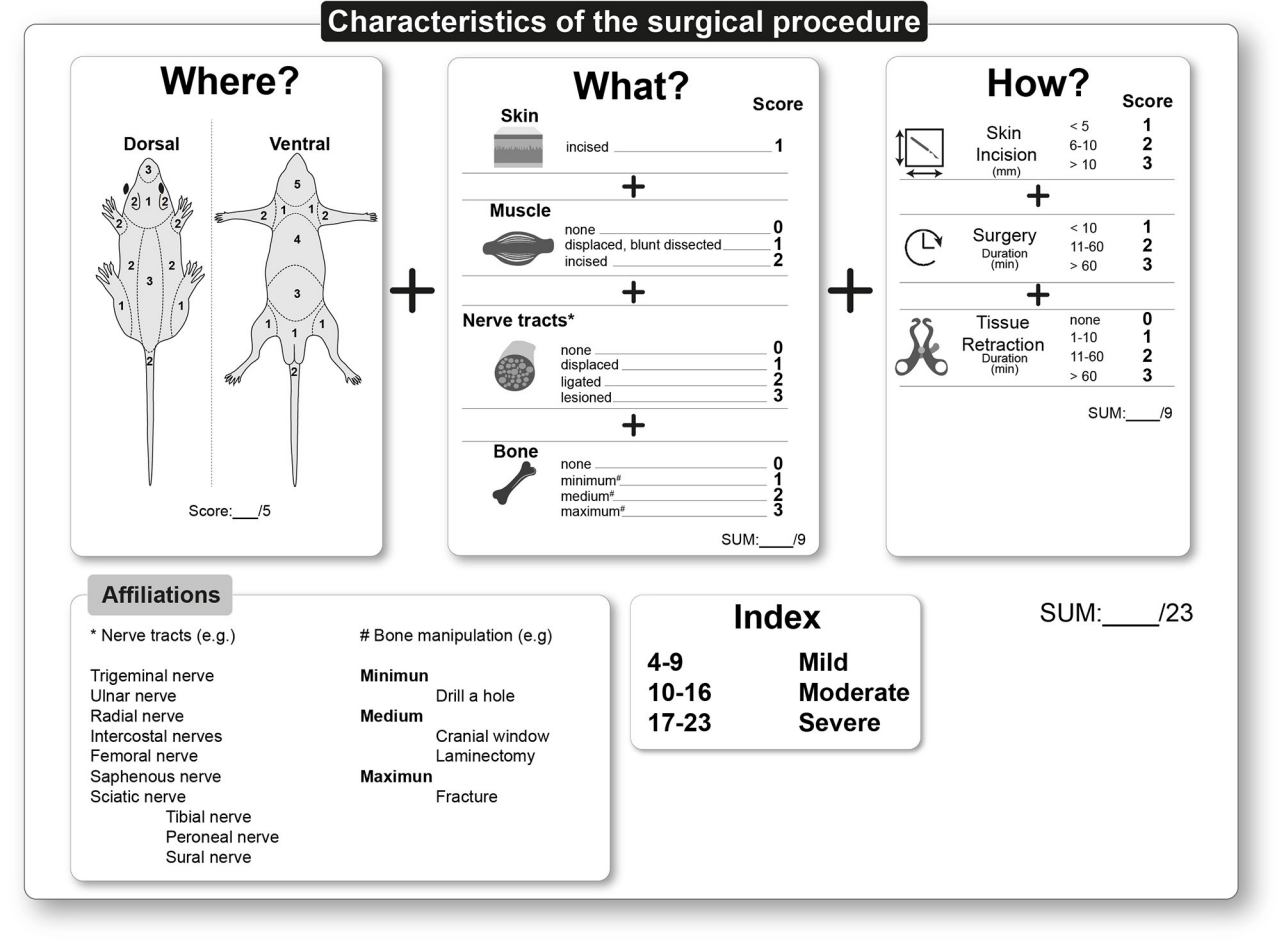
Classification chart for the prospective severity assessment for surgical procedures based on the WWHow concept. The surgical intervention is characterized in terms of “Where”, “What” and “How” and the corresponding scoring. The points are summed and calculated into a total score for the surgical intervention. The minimum score is 4; the maximum is 23. Procedures leading to a score between 4 and 9 result as mild, between 10 and 16 as moderate, and 17–23 points indicate a severe prospective severity assessment of the surgical procedure. (The illustration (“Where”) was created by modifying images purchased in the PPT Drawing Toolkits-BIOLOGY Bundle from Motifolio, Inc.).

In addition to skin and muscle trauma, sensory nerve fiber tracts are often displaced, crushed, ligated, or lesioned in some surgical procedures (9, 34–36). Injuries of small cutaneous nerves play a minor role, whereas large nerve fiber tracts, such as the sciatic, femoral, intercostal, radial, or ulnar nerves with their direct branches (e.g., first branches of the sciatic nerve: tibial, peroneal, and sural nerve, see Figure 2) are more relevant. The grade of nerve injury can significantly affect the post-operative severity. Therefore, to assess the potential severity, it is essential to know whether and how nerve fibers tracts are injured, where and in which body regions the neuropathic pain symptoms can be expected. Nerve trauma during surgeries mainly involves partial damage to peripheral axons through blunt trauma, including crush, stretching, perineural inflammation, compression, and scar formation, with entrapment of sensory fibers and/or neuroma formation. In general, partial damage to sensory axons during surgery results in spontaneous activity, a lower activation threshold, and an enhanced response to a stimulus (37). Hyperesthesia can be ascribed to enhanced sensitivity of non-interrupted but injured axons associated with spontaneous ectopic discharges by increased ion channel expression along the axon. In addition, the inflammatory response may alter gene expression in the dorsal root ganglion, which increases the synthesis of peripheral receptors that sensitize nociceptors (38).

The displacement of nerve tracts within the soft tissue results in minor pathological consequences and is rated as 1. However, inadequate anatomical knowledge, abnormalities, or surgical techniques that may result in a lesion during surgery are rated as 3. In this case, neuroplastic changes in the entire neuroaxis of pain are expected, resulting in possible nerve degeneration and thus an increased transition from acute to persistent post-surgical pain. Nerve ligation is graded lower than the lesion itself and therefore rated as 2.

Other scientific questions require surgical interventions involving mechanical distortion of bones. Mechanical bone distortion can range from craniotomy over laminectomy to experimental bone fractures. In addition, consideration must be given to which part of the skeleton is injured and to what extent is essential for *maintenance behavior*. Disturbing periosteum, cortical bone, or bone marrow can induce bone pain, depending on different pathological processes. This suggests that nociceptive fibers innervate all bone structural compartments (39–41). The periosteum has the highest density of fibers arranged as a mesh network, allowing detection of mechanical distortion (e.g., stretching of periosteum). Mechanical distortion, such as a fracture or drilling a hole, directly activates nociceptive fibers, but this depends on the surgical trauma extent. These traumas may be associated with a short-lasting sensation of sharp and localized pain experienced in the immediate recovery phase after anesthesia, followed by a more long-lasting dull, deep pain. Hours after bone trauma, osteoclasts, osteoblasts, and immune cells release pro-inflammatory mediators, creating an inflammatory environment close to the trauma, contributing to the peripheral sensitization processes. A distinction must be made regarding the stability of the bone injury. While removal of bone tissue (drilling a hole) and partial replacement (cranial window) represent stable injury, a fracture can be unstable. Due to the uniform nociceptive fiber bone distribution, the dimension of the bone injury is directly related to the activation. Since no direct studies address this question, we used the size of the bone damage as a parameter to generate a score. Bone trepanation is rated 1 (minimum), a craniotomy for implantation of a cranial window or a laminectomy is rated 2 (medium), and fractures are rated 3 (maximum) (Figure 2).

#### Characteristics of Surgical Intervention (“How”)

Surgical interventions contain various factors that can considerably influence the outcome and, consequently, the severity. In contrast to humans, only a few studies in surgical models exist on how intra-operative factors affect the severity in rodents. In total, three intra-operative factors have been identified for post-operative severity assessment in rodents; the size of the incision, the duration of the surgery, and the time of tissue retraction.

The dimension of the skin incision is directly related to the activation of cutaneous nociceptive fibers and resident immune cells, such as mast cells or δT cells (20, 21). Based on data from various surgical-induced pain models, a skin incision <5 mm is categorized as a 1, between 6 and 10 mm as a 2, and >10 mm as a 3. However, there are no specific data from animal studies describing the effect of incision size on pain behavior, especially since this information is given little or not at all in publication. Therefore, the data are taken from the description of the standardized post-operative pain models in rodents (8, 42, 43).

It is known from human studies that minimally invasive procedures reduce cutaneous hypersensitivity but do not contribute significantly to the overall reduction of post-operative pain (44, 45). This may be due to the minimally invasive procedure limiting the surgeon's vision and leading to additional tissue trauma (e.g., surgical neuropathy by nerve injury), directly impacting severity. Therefore, skin incision should be kept to a minimum, allowing the best performance of further surgical stages (17).

The second factor is the duration of a surgical procedure, which is related to the duration of the anesthesia, the resulting physiological changes, such as the local lack of oxygen in the injured tissue, loss of body temperature, and the inflammatory processes. Prolonged surgical duration is mainly associated with a more extensive or complex intervention. Therefore, three-time intervals were defined: Short interventions under 10 min [e.g., plantar incision models (43)] are rated 1, medium-length between 11 and 60 min (e.g., muscle retraction model, (30) are rated 2, and long duration surgeries over 60 min (e.g., thoracotomy models, (46) are rated 3.

The third factor is tissue retraction using surgical tools (forceps or retractors), which damage the tissue in several ways. Retraction of superficial tissues such as skin and muscles leads to oxygen deficiency, accompanied by tissue damage and even destruction (47). Also, nerve tracts can be displaced or lesioned with tissue retraction, resulting in increased inflammatory response (30). Potential manipulation of nerve tracts and undersupply by the retraction process are potentially time-dependent. Therefore, based on the characteristics of different surgical models in rodents, three-time intervals were defined, which differed in terms of post-operative outcomes and mechanisms. Retraction time from 1 to 10 min is rated 1, 11 to 60 min is rated 2, and over 60 min is rated 3 (see for examples “duration of surgery”).

Other intra-operative parameters have not yet been directly investigated in rodents. However, studies with surgical patients show that other interoperative factors like anesthetic technique, the surgical unit's experience, or open vs. laparoscopic are known (48).

## Exemplary Application of the WWHOW Concept

Several exemplary surgical interventions are presented here to demonstrate the categorization according to the WWHow concept. The resulting prospective severity scores are explained in detail for each intervention. It is important to note that the data and values, e.g., for the incision size or the surgery duration, are based on published studies/protocols and may vary depending on the individual protocol. In addition, all surgical interventions described herein generally require adequate personal training for performance, aseptic techniques, potential ventilation, and intra-operative monitoring. Finally, all surgical interventions are performed under general anesthesia, potential analgetic regime, sterile, and in some cases, ventilated conditions (17).

First, we describe representative surgically-induced pain models in rodents, which address different intra-operative characteristics according to the WWHow concept. The pain-related behavior of these models performed without analgesia treatment is the subject of many studies in the pain field and thus represents a basis (“worst-case scenario”) necessary to predict the possible severity outcome of a surgical procedure. Next, common exemplary surgical interventions widely employed in biomedical research will be explained, categorized, and when available, the severity assessment of relevant catalogs is mentioned.

### Surgical Models in Rodents

#### Incision Models

**Figure 3:**
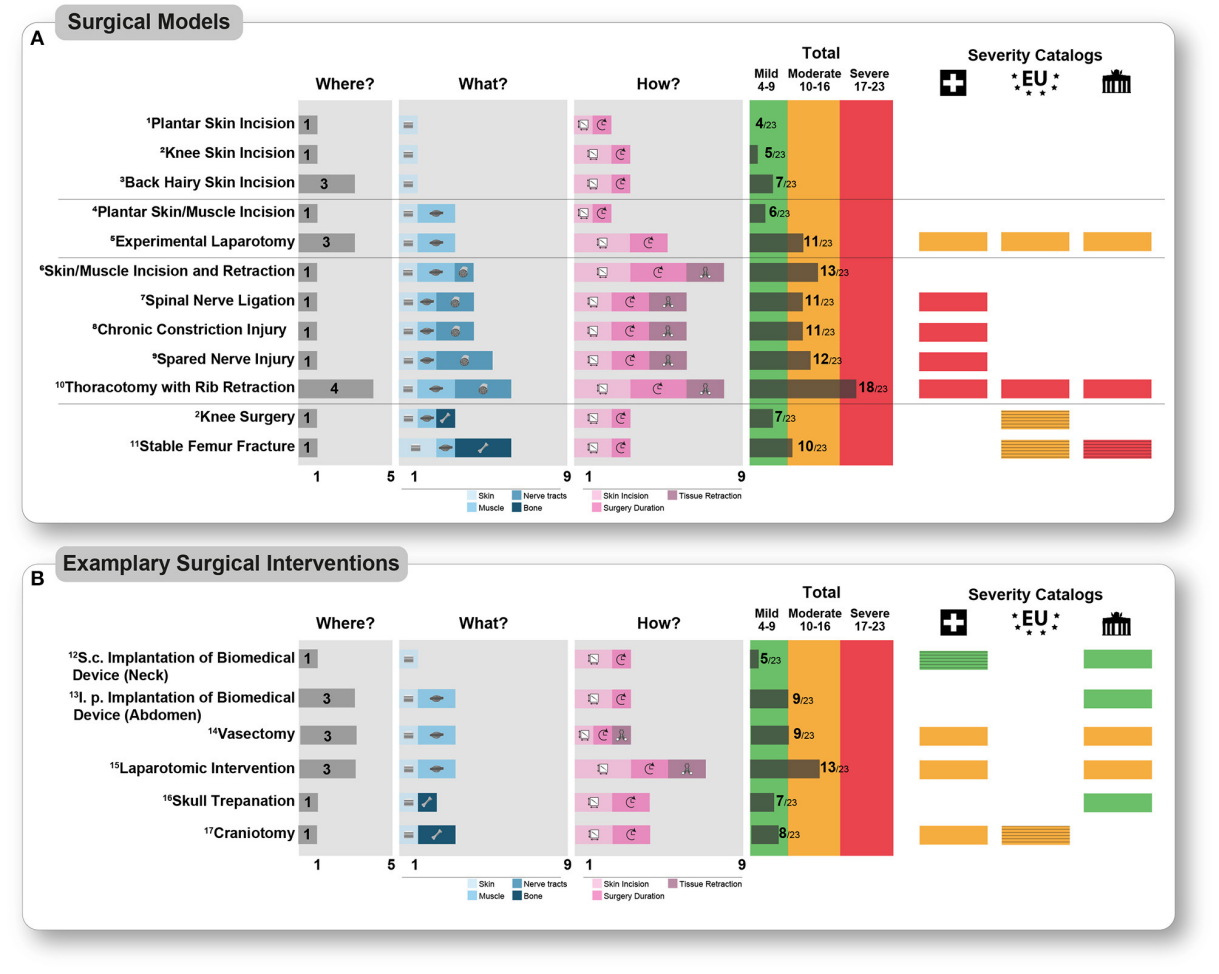
Detailed presentation of the scoring according to the WWHow concept of different pain models (A) and exemplary surgical interventions in biomedical research (B). The corresponding scoring is presented for each intra-operative parameter according to the WWHow concept. The individual factors are displayed pictographically for the “What” (blue shades) and “How” (magenta shades) categories according to the scoring. The total score is illustrated for each surgical intervention without any analgesia treatment and categorized into “mild” (green color, score 4–9), “moderate” (orange color, score 10–16), and “severe” (red color, score 17–23). On the right part of the figure, the severity assessment according to three widely used catalogs, namely the Swiss (Swiss symbol), the EU (EU symbol), and Berlin animal welfare (Brandenburger Tor symbol), are labeled in the corresponding colors if the interventions are listed there. The grading was represented by striped colors if a similar intervention was found. If no corresponding intervention was found in the catalogs, this position is white. ^1^Pogatzki and Raja (42); ^2^Buvanendran et al. (49); ^3^Duarte et al. (50); ^4^Brennan et al. (43); ^5^Kendall et al. (51); ^6^Flatters (30); ^7^Ho Kim and Mo Chung (52); ^8^Bennett and Xie (53); ^9^Decosterd and Woolf (54); ^10^Buvanendran et al. (46); ^11^Jimenez-Andrade et al. (55); ^12^Lu et al. (56); ^13^Ren et al. (57); ^14^Awsare et al. (58); ^15^Zieglowski et al. (59); ^16^Llovera et al. (60); ^17^Kyweriga et al. (61).

Skin incision is an intra-operative component of many surgical procedures. Accordingly, various models in rodents address this injury. These models differ mainly in the localization and dimension of the skin incision. The plantar incision model was established in the rat in 1996 and in the mouse in 2003 to study post-operative pain mechanistically (42, 43). The incision is made unilaterally on a hind paw, causing evoked pain-related behavior represented by hypersensitivity to mechanical and heat stimuli. In this model, a 1 cm longitudinal incision in rats, or a 0.5 cm in mice, of the glabrous skin and fascia in the plantar aspect of the hind paw under general anesthesia and sterile conditions is performed with a scalpel (no 11). Compresses stop the bleeding caused by the skin incision. The skin is closed with a mattress suture. The duration of the entire intervention is <10 min. Thus, after applying the WWHow concept for the category “Where”, 1 point, for “What” 1 point, and for “How” 3 points in rats, and 2 in mice because the different incision dimension (for the detailed calculation, see Figure 3 and Supplementary Figure 1A). This surgical intervention results in a total score of 5 points in rats and 4 points in mice, leading to the mild severity level categorization. Even if the incision size is different between mice and rats, this does not lead to a higher categorization (robustness to individual procedures) but may also depend on the animal's overall size.

Changes in the intra-operative characteristics, e.g., incision dimension in “knee skin incision model” (49), enhanced the score (Figure 3). Skin-only incision as a procedure is not clearly categorized in any of the severity catalogs. By comparing the tissue trauma (from pre-clinical models), these are comparable to incisional wounds, skin, and ear biopsies. This type of injury is categorized as mild in all catalogs, but without considering the injury's location.

A change in the incised body region affects the total score, as in the “back hairy model” (50), although the tissue trauma is equal. Based on the available data, which have explicitly investigated post-operative pain behavior leads to medium persistence evoked pain-related behavior, which is represented by hypersensitivity to mechanical and heat stimuli around the incision wound.

Postoperatively, animals exhibit pain avoidance behavior, e.g., guarding the incised paw, limping (antalgic gait) during locomotion (19, 29, 31, 62), or spontaneous pain behavior [e.g., foot lifting (63), grimacing (64, 65)]. Additional manipulation of the underlying muscle tissue, whether by blunt dissection or incision, results in hemorrhage and exacerbates guarding behavior (31).

#### Surgical Nerve Injury Models

Nerve injury can be an intentional or unintentional element of a surgical procedure. Therefore, it is essential to distinguish which nerve was damaged and how. To what extent the manipulation of epidermal cutaneous nerve fibers influences the severity of surgical intervention has been poorly studied so far. Therefore, we will focus on manipulating nerve tracts because models exist for this purpose. For over three decades, rodent surgical-induced nerve injury models have been developed to study neuropathic pain's molecular, cellular, and circuit mechanisms (9, 34, 35). Typical neuropathic pain models manipulate the sciatic nerve and its three branches; the tibial, peroneal, and sural nerve. Skin incision and blunt preparation of the underlying biceps femoris muscle are necessary to display the sciatic nerve with their trifurcation and ligate or lesion it according to the particular model.

A concrete example is the spared nerve injury (SNI) model (54). In the SNI model, two of the three branches of the sciatic nerve are ligated and lesioned, leaving the sural nerve intact. The intervention has an approximate total duration of 15 min. Overall, the post-operative severity is categorized as moderate based on the total score of 12. (“Where”, 1 point, “What” 5 points, “How” 6 points, in total 12 points (for the detailed calculation, see Figure 3 and Supplementary Figure 1B). Pain symptoms in this model are evolving mechanical and cold hypersensitivity in the lateral area of the paw, which is innervated by the spared sural nerve. Axotomy denervates tibial and peroneal dermatomes of the hind paw, resulting in total loss of sensory perception (hypoaesthesia) motion control. Around the skin incision above the biceps femoris muscle develops primary hypersensitivity to mechanical and heat stimuli (comparable to skin incision models). In addition, guarding behavior and an antalgic gait can be observed due to the mechanical stimuli caused by the injury in the musculus biceps femoris in the acute post-operative period. Only the swiss catalog explicitly categorizes this model and other surgical nerve injury models as severe. Other surgical nerve injury models have equally moderate severity (52, 53). Other severity catalogs categorize these interventions only indirectly or not at all (Figure 3).

Next to manipulating the sciatic nerve, there are other possibilities in which nerve tracts can be damaged or injured. As in the “skin/muscle incision and retraction” model (30), displacing nerve tracts by soft tissue retraction does not result in direct nerve injury, but a prolonged pain-related behavior is presented. In addition, direct nerve damage can occur when nerves are mechanically compressed against rigid structures such as the bone. An example of this is the “thoracotomy with rib retraction (TRR)” model (46). Thoracotomies are necessary for various models to, e.g., study myocardial diseases, such as myocardial infarction or heart insufficiency. Practically, a relative to the body size of the rodent, large incision between the 4 and 5th ribs around 3–4 cm skin and an ~1.5 cm muscle and pleura incision are performed in the TRR rat model. The incision is likely to cause hemorrhage of both the skin and the muscle layer. A small self-retaining retractor opened the intercostal space for possible inner body interventions of 60 min. Because of retraction, intercostal nerves are lesioned by compression to rigid structures, here the ribs. The intervention has an approximate total duration of 90 min. Overall, the severity after awakening from anesthesia is categorized as severe based on the total score of 18 (“Where”, 4 points, “What” 6 points, and “How” 8 points, for the detailed calculation, see Figure 3 and Supplementary Figure 1A). The typical post-operative pain symptoms are similar to other surgical models involving nerve injury, especially non-evoked pain in the territory of the intercostal nerves. It has been shown that significant intraindividual differences exist in the intensity of pain-related behavior that is not due to the extent of nerve injury but to the complexity of the surgical intervention itself. The extent of post-operative rodent-specific and observable behaviors change after awakening from anesthesia has not yet been studied systematically and requires further investigation. Based on the findings from other pain models, it can be interpolated that the animals have restrictions in *movement behavior* (climbing, rearing, walking), which is caused by the surgical wound at the thorax region. The severe severity assessment by the WWHow-concept is in line with other severity catalogs (Figure 3).

### Bone Injuries

In many biomedical experiments, manipulation of the skeletal system is a component, such as implanting catheters or optical fibers in spinal and supraspinal structures or generating a disease state. Orthopedic and fracture pain models are particularly worth mentioning here, as various have been developed and established recently (41).

#### Skull Trepanation and Craniotomies

Skull trepanation represents an essential technique in neuroscience to directly access the brain, whether for injection or implantation of optical fiber or infusion devices. Craniotomies are more invasive than trepanation and are necessary, for example, to implant a cranial window or multifiber devices into the brain. As an example, here, we determine the severity of a skull trepanation (60): The scalp is incised by scalpel dorsally between 0.5 and 1.5 cm, exposing the bregma and the relevant skull region. Muscles or nerve tracts are not present there. A hole is drilled into the skull; all bone layers are injured. However, the underlying dura mater is not traumatized. Stereotactic micro-injection is performed with a thin needle or glass capillary (usually 30G or smaller) through the dura mater after a scalp suture. A surgery duration from 10 to 60 min is plausible depending on the injection location, volume and injection speed, and reagent to be injected. Tissue retraction is not necessary or feasible. Thus, after applying the WWHow concept for the category “Where”, 1 point, for “What” 2 points, and for “How” 4 points (for the detailed calculation, Figure 3 and Supplementary Figure 1B). This surgical intervention results in a total score of 7 points (Figure 3), leading to the mild severity level categorization. Cutaneous mechanical and heat hypersensitivity is expected postoperatively (compared to plantar incision model), associated with localized bone pain around the trepanation. Because the dorsal side of the skull is not subjected to any other direct external mechanical stimulation, e.g., movement; bone pain, and the associated severity is relatively short and anatomically less relevant for the rodent and will decrease with healing (up to 2 days). Severity catalogs also classify skull trepanation as mild (Figure 3). Compared to trepanation, craniotomies usually do not differ in severity assessment, but the bone defect is larger (61). The extent to which such interventions impact severity has not yet been established. This increases the “What” score to 2 or 3, but the overall scoring (8–9) does not change the mild score.

Various models have been developed to mimic orthopedic surgery and bone fractures to study the consequences of skeletal surgery in a patient-oriented approach. Contrary to procedures on the skull, these are mainly performed on the bony locomotor apparatus and are associated with more significant bone trauma. For example, a 1 cm skin incision is prepared over the patella tendon in rats. The tendon is disengaged from the fascia, and, using a diamond drill, a 1.4 mm diameter and 0.5 mm deep hole with a total surgery duration of 5 min is performed (49). Applying the WWHow concept for the category “Where”, 1 point, for “What” 3 points, and for “How” 4 points are given (Figure 3). This surgical intervention results in a total score of 7 points (Figure 3), which leads to the categorization in the mild severity level. Cutaneous mechanical and heat hypersensitivity is expected postoperatively (compared to the plantar incision model), associated with localized bone pain around the holes. Changes in rodent-specific behavior, such as rearing and ambulation, are observed up to 3 days postoperatively. Swiss and the EU catalogs assign moderate to similar surgical interventions.

### Laparotomic Interventions

Laparotomies are conducted to perform implantations, visceral organ harvesting, manipulating, or ectomies. A male mouse vasectomy (58) is described here as an example of a laparotomy procedure: The abdomen's left and right inguinal region is opened with an incision (<0.5 cm) underlying muscle layer. Next, the spermatic duct is ligated twice and dissected between the sutures. Finally, the muscle layer is closed by sutures, and the skin is restored with staples or sutured. Depending on the exact surgical procedure, such a procedure lasts <10 min with minimal tissue retraction using a small metal retractor. Thus, after applying the WWHow concept for the category “Where”, 3 points, for “What” 3 points, and for “How” 3 points (Figure 3 and Supplementary Figure 1B), resulting in a total score of 9 points, which leads to categorization in the mild severity level. This categorization is in line with the swiss catalog.

In contrast, an experimental model for laparotomy (51) totals in a score of 13 and would thus have a moderate severity score because of a larger skin and muscle incision (2 cm) (Figure 3 and Supplementary Figure 1A). Again, hemorrhage of both the skin and muscle layer must be expected. Cutaneous mechanical and heat hypersensitivity are expected postoperatively, directly around the suture up to 72 h. Rodent-specific behaviors, such as grooming and nesting, are reduced by laparotomy in the acute post-operative period (up to 36 h) because the abdominal muscles necessary for these behaviors are injured (66). Here is a difference in severity between the plantar incision and the experimental laparotomy model. The characteristics of the tissue trauma are similar. However, incision localization, size, and the prolonged surgery duration categorize the experimental laparotomy (13/23 p), in contrast to the planar incision (6/23 p), in the moderate severity category. A moderate severity level was also suggested in other severity catalogs (Figure 3).

## Discussion

The prospective severity assessment of an animal experiment is a significant component of experimental planning. It is mandatory to evaluate an experiment's ethical justifiability and knowledge gain (harm-benefit analysis) as stipulated in the Directive 2010/63/EU. Three main catalogs are available in the EU for prospective severity assessment and can provide rough guidance (4–6). However, in many cases, classifications in these catalogs are mainly expert and experience-based and only partly evidence-based. Furthermore, these catalogs are primarily lexicon-like, without giving the possibility of adaptation to the particular experiment. Consequently, we aimed to provide a conceptual framework, termed “WWHow-concept”, based on data from surgical-induced pain models in rodents and allows the determination of the severity categories (minor, medium, and severe) in a prospective, adaptable, and transparent manner.

### Animal Welfare Science With the Help of Pain Models in Rodents?

In patient-oriented pain research, animal pain models are used to investigate the mechanisms of pain diseases or pain symptomatology. The most commonly used species to study pain are rats and mice (11, 35, 67). Using established rodent pain models provides a framework to objectively describe the “worst-case scenario” of animal well-being changes resulting from tissue damage and other interventions (8, 9, 12). This scenario can assist in defining a generally valid zero point for critically reviewing peri-operative refinement actions in terms of their impact. Notably, the available models address a wide range of different pain entities, ranging from acute substance irritation-related pain to tumor-related pain or inflammatory pain, to name a few (8, 34, 35). Behavioral tests are applied to measure the intensity and quality of the pain directly. Pain-related behavior tests in rodents are primarily performed without analgesia treatment. The underlying mechanisms of pain, or the developments of new analgesic compounds, are the scientific question of many studies in this field (34, 35). Notably, the thorough investigation of diverse aspects of the pain-related behavior in the different models (68–70) provides an essential prerequisite for the development and characterization of new analgesic targets. The available results simulate a worst-case scenario in which the direct effect of various pain modalities on the rodent is documented in a time-dependent manner under standardized laboratory conditions. Our approach did not require any additional animal experiments.

### The WWHow Concept—A Chance for Prospective Severity Assessment?

We propose a categorized, easily understandable, and applicable, transparent approach for a prospective severity assessment of rodent surgical interventions, which relates to the specific anatomy and behavior and biomechanical properties. The localization of surgical intervention and the associated influence on pain nature and well-being are enormous (66, 67). This has been shown in many rodent studies with surgically-induced pain models and the investigation of rodent-specific behaviors, such as grooming (66), burrowing (71) or nest building (72, 73). Based on the surgical intervention site, specific tissue traumas are associated with developing different pain natures, their intensity, and duration. The underlying mechanisms are reported in many studies, depending on the respective tissue trauma, and, thus, provide the basis to prospectively name the occurring types of pain in terms of duration, intensity, and localization in an evidence-based manner (10). Knowledge about the effects of surgical interventions in rodents will help avoid scientifically incorrect and potentially fatal analogies from humans to rodents.

### Comparison With Severity Catalogs

This approach has several advantages compared to the most commonly used severity catalogs (Figure 3). Catalogs list and describe surgical interventions with varying levels of detail and simply categorize the severity assessment without further explanation. It is, therefore, challenging to extrapolate individual or non-listed models based on the catalog considerations. The WWHow severity categorization is transparent, adaptable, and based on considerations (Where, What, How), not represented in other guidelines or catalogs.

Severity catalogs provide a valuable resource for the researcher and the regulatory/ permitting agency to categorize the severity. This is in a first step, a prospective process. Catalogs can only be considered a collection of different interventions and, thus, represent a first rough orientation aid, which must be supplemented in each case by an individual assessment, which is also required in the EU directive. The score presented here with the WWHow concept represents a module, based on intra-operative characteristics, estimates the maximum severity without analgesic treatment to be expected prospectively.

Moreover, in contrast to the catalogs, transparency is highly increased. It was possible to directly compare several interventions with severity catalogs and the WWHow concept. Critically, except for the EU catalog, it is not apparent whether or which analgesia regime was or was not considered in the other two catalogs. For laparotomy procedures, as well as for thoracotomy, the same categorization could be found. There were differences in the stable femur fracture, whereby an exact comparison between the surgical model and the description from the catalog was only possible to a limited extent. In addition, vasectomy was classified as moderate by both the Swiss and Berlin catalogs, whereas the application of the WWHow concept prospectively predicts mild severity. Additionally, empirical findings from, e.g., score sheet evaluation (59) and/or behavioral testing, home cage monitoring (74) show that the prospective severity score may be overestimated from a retrospective point of view, but this could be case dependent and not generalized. Corresponding detailed prospective and retropspective studies would enable clearer evidence.

It should be noted that vasectomy, according to the WWHow concept, can also be classified as moderate if other surgical protocols are used. This fact underlines the potential of the modular construction of this severity assessment using the WWHow concept, which is the separate consideration of each intervention as required by the EU Directive. Differences between the WWHow concept and the catalogs were in the surgical-induced neuropathic pain models, which are concretely assessed only in the Swiss catalog. In the other catalogs, no corresponding interventions could be indexed. All three neuropathic pain models (Figure 3) are classified as moderate by the WWHow concept.

In contrast, the Swiss catalog classifies them as severe. The classification with the WWHow concept shows that the severity is mainly determined by the nerve injury, which is distinct across the models. While in the “spinal nerve ligation” and “chronic constriction injury” models, there is ligation of the entire sciatic nerve, in the “spared nerve injury” model, there is a transaction of two sciatic nerve branches. Both procedures cause different (location and duration) pain symptoms, reflected by the WWHow concept. The Swiss catalog does not differentiate here, and the user cannot understand the classification due to a lack of transparency. However, knowing what tissue trauma looks like and how it occurs is needed to address prospective refinement actions that can potentially minimize severity directly. In addition, this knowledge can contribute to a fact-based dialogue with authority officials and other stakeholders. Importantly, future publications, harboring surgical interventions, should consider the WWHow concept for validation thereby not only confirming the scoring concept but also allowing the expansion of methods in this technically fast growing scientific century.

### Limitations

This concept is based on surgical interventions—the procedure itself. The influence of the rodent strain, genetic manipulation effects, potential sex or age differences are not considered (27). Similarly, preoperative factors as particular treatments or behavioral-test induced stress or housing factors are not considered (11). Therefore, it is essential to strengthening the fact that the WWHow concept is applicable solely for surgical interventions as one part of a whole experimental procedure. Other experimental parts like behavioral tests, particular pre-treatments, and post-operative investigations must be considered separately to categorize an entire animal experiment.

### Outlook

The WWHow concept may serve as a core module and should be included in future prospective studies. Moreover, the concept constitues a basis for further categorization attempts to enable the transparent, adaptable, and prospective categorization of various animal experimental procedures and thereby enable their refinement. In the future, it would be helpful to establish concepts for classifying animal tests that include behavioral tests as such and in combination with specific treatment options. Moreover, due to the existing knowledge gaps mentioned in the limitations, the scoring system presented here is initially a first conceptional framework that should be regularly updated and expanded. In addition, the WWHow concept provides the basis for the development of recommendations for anesthetic and analgesic management and the preparation of experiment-related score sheets to evaluate actual severity concerning peri-operative characteristics. Thereby, our concept not only constitutes an accessible and broadly usable scoring system, it also present a system with potential for refinement strategies.

## Data Availability Statement

The original contributions presented in the study are included in the article/Supplementary Material, further inquiries can be directed to the corresponding author/s.

## Ethics Statement

Ethical review and approval was not required for the animal study because reterospective results of published studies were evaluated. No animals were used for this study.

## Author Contributions

AT-T, CP, and DS developed the score initially, finalized it with all other authors, and designed the figures. DS implemented the designed figures. AT-T and DS wrote the manuscript. The manuscript and its results were discussed with all authors. All authors listed have made a substantial and intellectual contribution to the work and approved it for publication.

## Conflict of Interest

ND is an external consultant and animal welfare officer at Medizinisches Kompetenzzentrum |c/o HCx Consulting GmbH | Ulmenstraße 12 | 15864 Wendisch Rietz During the last 5 years, EP-Z received financial support from Mundipharma GmbH and Grunenthal for research activities and from Grunenthal, MSD Sharp & DOHME GmbH, Mundipharma GmbH, Mundipharma International, Janssen-Cilag GmbH, Fresenius Kabi, and AcelRx for advisory board activities and/or lecture fees. The remaining authors declare that the research was conducted in the absence of any commercial or financial relationships that could be construed as a potential conflict of interest.

## Publisher's Note

All claims expressed in this article are solely those of the authors and do not necessarily represent those of their affiliated organizations, or those of the publisher, the editors and the reviewers. Any product that may be evaluated in this article, or claim that may be made by its manufacturer, is not guaranteed or endorsed by the publisher.

## Supplementary Material

Supplementary Figure 1 Exemplary detailed scoring for different surgical interventions in rats **(A)** and mice **(B)** according to the WWHow concept, showing the respective scoring points of the “Where”, “What” and “How” categories and the total score from which the severity is determined.
