# Supplementary figures and images for: The “WWHow” Concept for Prospective Categorization of Post-operative Severity Assessment in Mice and Rats
Source: Front Vet Sci. 2022 Mar 15;9:841431. doi: 10.3389/fvets.2022.841431 (PMC8964947; doi:10.3389/fvets.2022.841431)

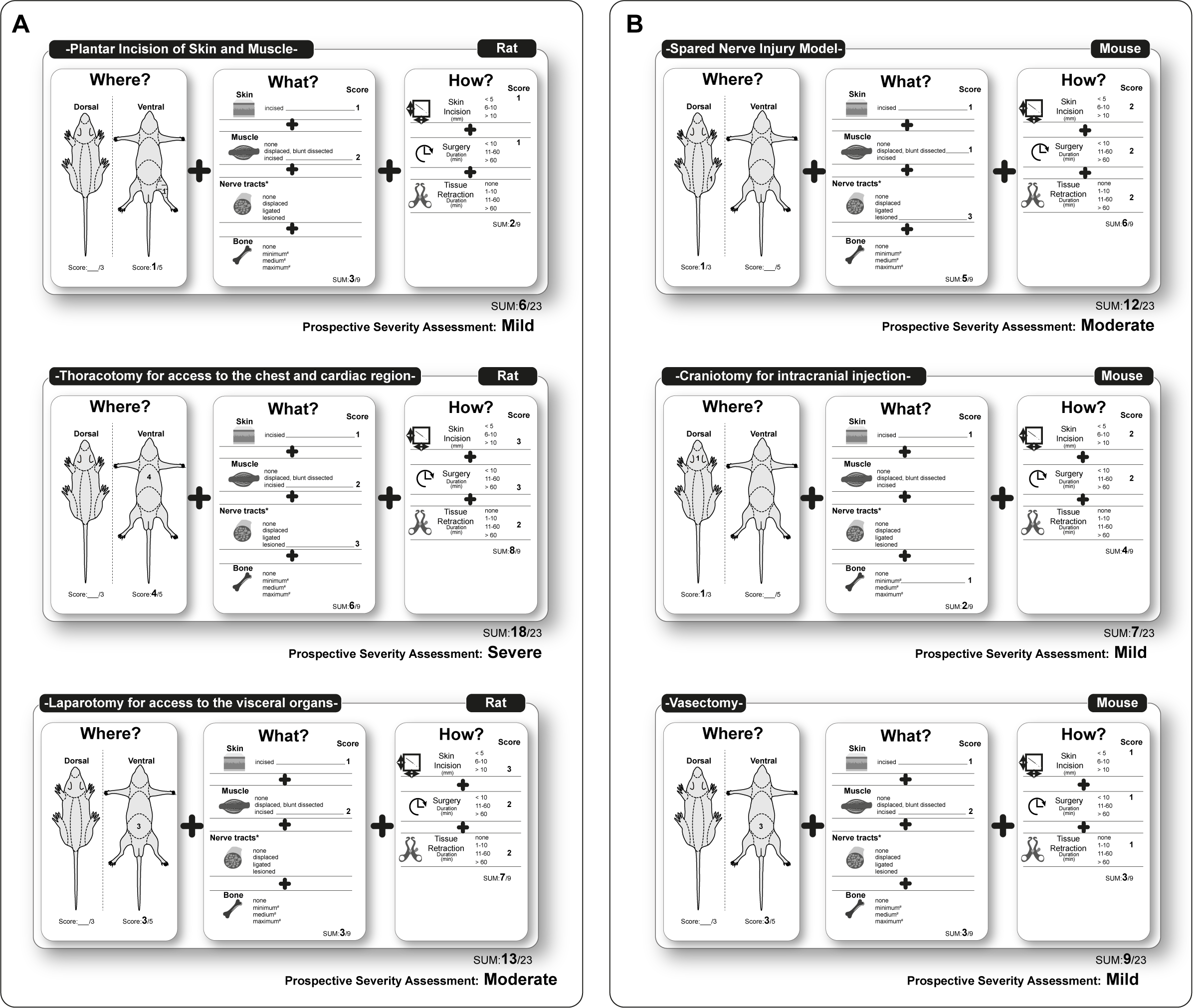

Supplement: Supplementary Figure 1 — Exemplary detailed scoring for different surgical interventions in rats (A) and mice (B) according to the WWHow concept, showing the respective scoring points of the “Where”, “What” and “How” categories and the total score from which the severity is determined. [file Image_1.TIF]
